# Single-Crystal Diamond Needle Fabrication Using Hot-Filament Chemical Vapor Deposition

**DOI:** 10.3390/ma14092320

**Published:** 2021-04-29

**Authors:** Rinat Ismagilov, Sergei Malykhin, Aleksey Puzyr, Artem Loginov, Victor Kleshch, Alexander Obraztsov

**Affiliations:** 1 Department of Physics, M.V. Lomonosov Moscow State University, 119991 Moscow, Russia; Sergeim@uef.fi (S.M.); loginov.ab15@physics.msu.ru (A.L.); klesch@polly.phys.msu.ru (V.K.); obraz@polly.phys.msu.ru (A.O.); 2 Department of Physics and Mathematics, University of Eastern Finland, 80101 Joensuu, Finland; 3 Division of Solid State Physics, Lebedev Physical Institute of the Russian Academy of Sciences, 119991 Moscow, Russia; 4 Federal Research Center “Krasnoyarsk Science Center SB RAS”, Russian Academy of Sciences, Institute of Biophysics, 660036 Krasnoyarsk, Russia; apuzyr@mail.ru

**Keywords:** thin films, diamond needles, chemical vapor deposition, hot-filament CVD, large-scale synthesis

## Abstract

Single-crystal diamonds in the form of micrometer-scale pyramids were produced using a combination of hot-filament (HF) chemical vapor deposition (CVD) and thermal oxidation processes. The diamond pyramids were compared here with similar ones that were manufactured using plasma-enhanced (PE) CVD. The similarities revealed in the morphology, Raman, and photoluminescent characteristics of the needles obtained using the hot-filament and plasma-enhanced CVD are discussed in connection with the diamond film growth mechanism. This work demonstrated that the HF CVD method has convincing potential for the fabrication of single-crystal diamond needles in the form of regularly shaped pyramids on a large surface area, even on non-conducting substrates. The experimental results demonstrated the ability for the mass production of the single-crystal needle-like diamonds, which is important for their practical application.

## 1. Introduction

Diamond has a number of attractive properties, including high electron and hole mobility, the highest known thermal conductivity, a wide energy bandgap, optical transparency window spanning from the near ultraviolet to the far infrared, and chemical inertness, which make it a unique and desirable solid-state material for several forefront technological applications [1,2,3,4,5,6,7,8]. In particular, diamonds with elongated shapes are required for quantum optical sensors [9]. Chemical vapor deposition (CVD) is nowadays considered to be one of the most suitable methods for fabricating diamond films. In our previous publications, we showed that polycrystalline diamond films composed of a mixture of micrometer-scale single crystals of pyramidal shape and highly disordered carbon material may be produced via CVD using a hydrogen–methane gas mixture that is activated by a direct current discharge (DC CVD) with appropriately chosen process parameters [10]. The diamond crystallites may be extracted from the film via selective oxidation, which allows for the gasification and removal of the disordered carbons from the CVD films. The crystallites produced by the combination of DC CVD and selective oxidation have perfect pyramidal shapes and a radius of curvature at their apex in the range of a few to a hundred nanometers. The effects of the DC CVD process parameters on the shape and properties of the needles were investigated [11,12,13,14,15], as well as the ability for the controllable creation of luminescent color centers, such as silicon-vacancy (SiV), nitrogen-vacancy (NV), and germanium-vacancy (GeV) color centers, during the single-step growth process. One of the limiting factors of the DC gas discharge-based technology is the necessity for conductive substrates for the film deposition.

In the present study, we developed an alternative diamond needle fabrication route that involved the combination of the hot-filament CVD (HF CVD) technique and a thermal oxidation process. The choice of using the hot-filament CVD method instead of plasma-enhanced CVD was dictated by the desire to develop a more simple, more efficient, and large-scale diamond needle production method, which eliminates the need for utilizing complex molecular plasma. Here, we demonstrate the successful fabrication of the single-crystal needle-like diamonds using HF CVD, which opens up new prospects for their practical usage.

## 2. Materials and Methods

**Figure 1:**
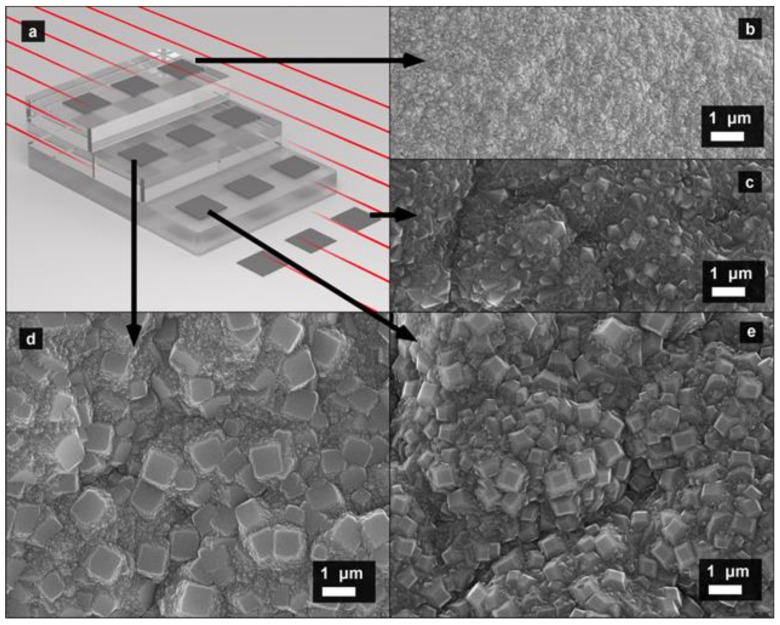
Typical scanning electron microscopic images of polycrystalline diamond films obtained using hot-filament chemical vapor deposition: (**a**) schematic illustration of the substrate holder configurations used for the synthesis of different diamond films in hot-filament chemical vapor deposition: three semitransparent gray tiles represent a stair-like quartz substrate holder, the 12 smaller dark gray squares are Si substrates, and the red lines are hot filaments of the HF CVD system; (**b**–**e**) SEM images for samples grown on substrates with a different location, indicated by arrows.

A hot-filament CVD reactor (HF CVD Model 655, Sp3 Diamond Technologies, Santa Clara, CA, USA) was used for the diamond film production. The reactor was equipped with 31 straight tungsten filaments (Sp3 Diamond Technologies, Santa Clara, CA, USA). This allowed for thin-film deposition on flat substrates with an overall diameter of up to 305 mm (see [16,17] for details). In order to inspect a wider range of deposition parameters, a special stair-like quartz substrate holder was used for the diamond film growth. A sketch of the stair-like quartz substrate holder, tungsten filaments, and Si substrates is shown in Figure 1a. The temperature of the Ø 0.127 mm straight W filaments that were heated using a DC was measured through a quartz window using a two-color pyrometer. The working parameters of the CVD system were established to maintain a filament temperature of approximately 2300 °C. The gas mixture pressure and total hydrogen–methane gas flow rate were maintained at 5.5 Torr and 822 sccm, respectively. The methane concentration was set to be equal to 2.7%. These conditions provided substrate temperatures in the range from 740 to 800 °C with equal 20 °C increments. The temperature of 740 °C corresponded to substrates farthest from the hot filaments. The growth duration was about 8 h.

Prior to the deposition, 20 × 20 mm^2^ polished mirror-like Si (100) substrates with a 0.5 mm thickness were washed in ethanol before placing a few droplets of the ethanol suspensions of detonation nanodiamonds on their surface. The detonation nanodiamond suspension was made by adding ethanol to the highly stable aqueous suspension of nanodiamonds (see [18,19] for the nanodiamond details). The ethanol addition led to a deterioration of the colloidal stability of the resultant suspension and aggregation of the nanodiamond particles. Finally, the Si substrates were dried on a spin drier at 1000 rpm for 1 min. The Si substrates were located on the stair-like holder (made from quartz plates) in such a way that the distance to the hot filaments on a particular shelf was fixed (see Figure 1a).

In order to perform thermal oxidation, samples of the HF CVD films were heated in an oven in air under normal pressure.

The Raman and photoluminescent (PL) spectra of both pristine and oxidized samples were studied at room temperature using a U-1000 (Jobin Yvon, Montpellier, France) Raman spectrometer with excitation at the 514.5 nm line of an Ar-ion laser. The scanning electron microscopy (SEM) measurements were performed using an LEO 1550 (Zeiss, Oberkochen, Germany) microscope that was operated at 5 and 15 kV accelerating voltages. The substrates with carbon deposits, both as-grown and after selective oxidation, were directly studied using SEM without preliminary preparation.

## 3. Results and Discussion

Figure 1 shows typical SEM images of the HF CVD films grown on the substrates located on different steps of the quartz stairs. The SEM inspection revealed that some of the obtained samples were presumably composed of disordered material (with the sizes of its structural elements below the SEM resolution limit) and crystallites with a micrometer size came out and shone on the surface. Similar SEM images are typical for polycrystalline diamond samples obtained using plasma-enhanced CVD [20], which were comprehensively studied in previous experimental and theoretical works [21,22]. In accord with these previous investigations, it may be assumed that the obtained HF CVD samples were composed of micrometer-sized diamond crystallites surrounded by disordered carbon material, which may have included amorphous, graphitic, and nanodiamond fractions.

**Figure 2:**
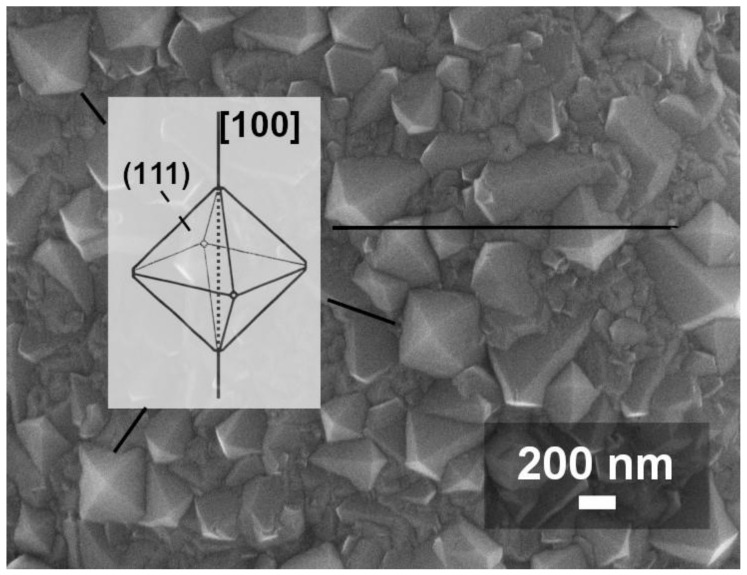
Typical SEM image of polycrystalline diamond films with predominantly triangular (111) faces, which were obtained on substrates that were the farthest from the hot filaments. The inset shows one of the two limiting shapes for an ideal single-crystal diamond based on growth along the <100> and <111> crystal directions. See the text for details.

The outer surface side of the polycrystalline diamond films, which were grown on substrates located at a maximum distance from the filaments (see Figure 1c and Figure 2), possessed a predominantly (111) grain texture with pronounced equilateral triangle facets, which were assembled around a <100> fourfold rotational symmetry direction. In these types of films, it is highly likely that all (100) grain facets had disappeared, degenerating to points lying on the fourfold symmetry axis. Hence, according to the growth mechanisms of diamond needles [23], there was no room for pyramidal needles with a (100) flat base. A similar needleless situation took place for the films, which were grown on substrates located at a minimal distance from the filaments. No flat grains or other needle traces were found using SEM. Similar SEM inspection procedures were applied to all obtained samples in order to exclude samples that were free from diamond needles. Further investigations in the framework of this study were made only for the polycrystalline films that presumably contained embedded single-crystal needles.

The as-grown films were oxidized by heating them in air at normal atmospheric pressure using a tube oven. The oxidation temperature and time were chosen to be 580 °C and 22 h, respectively. The material remaining on the Si substrate after the oxidation looked like a white-colored powder to the naked eye. This powder was easily detached from the substrate.

**Figure 3:**
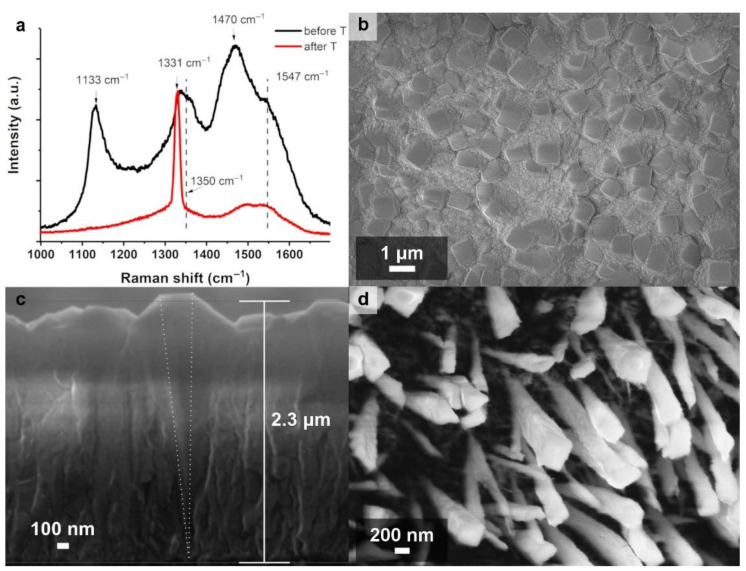
Typical (**a**) Raman spectra and (**b**–**d**) SEM images of the obtained diamond films. Black spectrum in panel (**a**) (indicated also as “before T”) and the images in panels (**b**,**c**) are as-grown CVD films. Red spectrum in panel (**a**) (indicated also as “after T”) and the image in panel (**d**) are for the same CVD film after thermal oxidation during 22 h in air at 580 °C. The median thickness of the as-grown diamond film and the location and spatial orientation of the needle (dotted lines) are approximately indicated in cross-sectional SEM image (**c**).

After the thermal oxidation, SEM inspections of the polycrystalline diamond films grown on the substrates that were located on the middle stairs during the HF CVD confirmed the presence of the pyramidal needles (see Figure 3). As seen in Figure 3c, the crystallites had sharp apexes and typically rectangular basal planes. The crystallite length varied from a few hundreds of nanometers to several micrometers, depending on the CVD process duration. The typical needle growth rate, which was determined via estimation of the film thickness using SEM images, was in the order of 500 nm/h. This growth rate is rather low in comparison to the 1000 nm/h that is achievable for diamond needles grown using plasma-enhanced DC CVD [13]. At first glance, a low formation rate may seem like a disadvantage; however, a low formation rate opens up the possibility for fine-tuning the growth process, which is necessary, e.g., for impurities introduction, morphology, and structural modifications of the diamond needles. Moreover, commercially available plasma-enhanced CVD systems usually offer a limited deposition area (up to 100 cm^2^), which provides homogeneous material growth, while hot-filament CVD has no such restriction (for example, the maximum deposition area of the setup used for this study was around 3000 cm^2^). A detailed inspection procedure suggested that the formation of a diamond film with embedded needles followed Van der Drift’s [24] competitive growth model, as explained in [21].

Figure 3 shows typical SEM images and the first-order Raman spectra of the obtained films before and after the thermal oxidation. The Raman spectra of the films demonstrated a strong diamond line peak at 1331 cm^−1^. The presence of other carbon species in the film was also indicated by the Raman spectrum as the broad bands centered at 1133 cm^−1^ and 1470 cm^−1^ for the nanodiamonds and 1350 cm^−1^ and 1547 cm^−1^ for the graphitic fraction [25,26,27]. The graphitic and nanodiamond materials were located between the microdiamond grains, as in the case of their deposition using plasma-enhanced CVD [28]. As seen in Figure 3, the thermal oxidation for 22 h in air at 580 °C was suitable for the selective removal of nondiamond phases from the thin polycrystalline film. Structural changes during this selective oxidation were successfully captured in the Raman spectra, which reflected the shrinkage of the diamond band and disappearance of the nondiamond bands. It is generally accepted that such a diamond line width reduction is related to diamond quality improvements, which, in our case, was achieved by etching the most defective crystallites and by a reduction in the mechanical stress induced by the nanodiamond phase [10].

**Figure 4:**
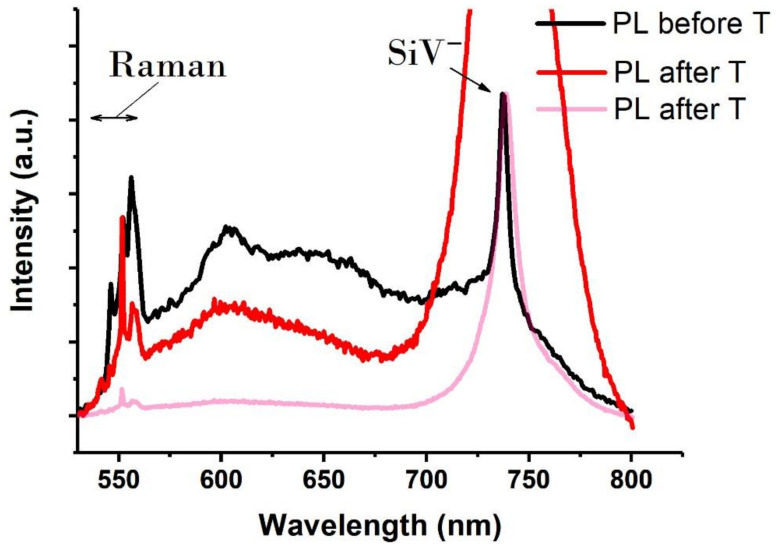
Typical photoluminescent (PL) spectra of the obtained diamond films. The spectrum obtained for the as-grown sample (PL before T) is shown as a solid black line. The spectrum obtained after heating this sample for 22 h in air at 580 °C (PL after T) is shown in two variants that differ in normalization. The spectra in pink and black were normalized using the intensity of the SiV^−^ line, while the spectra in red and black were normalized using the intensity of the diamond’s Raman line at 1331 cm^−1^. The spectral ranges corresponding to the Raman signal and the PL of the SiV centers are indicated by arrows.

Typical PL spectra obtained for the as-grown samples and for the same samples after their thermal oxidation are shown in Figure 4. As a rule, all these spectra contained Raman signals, as well as the intensive line located close to 738 nm. The position and shape of this line corresponded to the zero-phonon luminescence of the negatively charged SiV color center in diamond [15]. Similar to the case of the diamond needles obtained using DC-discharge plasma-enhanced CVD [15], the formation of the SiV centers may be explained by silicon substrate etching in an activated (ionized) hydrogen atmosphere and the subsequent introduction of gasified silicon precursors into the diamond lattice [29]. Additionally, silicon impurities in the diamond may have resulted from the chemical interaction of activated hydrogen with quartz (i.e., SiO_2_) elements (substrate supporting plates) in the reactor chamber. Since HF CVD does not explicitly imply plasma or free-electron generation, the mentioned growth mechanisms, which were realized during the DC-discharge plasma-enhanced CVD of the needles [15], could not be directly applied to this case. However, some similar chemical processes might be involved related to this concern, as follows.

The activated (atomic or ionized) hydrogen is generated in the HF CVD system and plays a crucial role in activating the different gas mixture processes, including initiating interconversions between CH_x_ and C_2_H_y_ species families (see, e.g., [30], where these conversions are discussed and their distribution map for the H atom mole fraction in an HF CVD system is calculated). Moreover, the evidence of the Si(100) surface etching by H atoms was observed previously [31]. Hence, it is natural to suppose that the etching of a Si substrate by energetic H atoms leads to the formation of volatile Si-based species (might be in the form of silane SiH_4_ or SiH_x_ radicals), which might participate in Si atom incorporation into the growing diamond lattice during HF CVD and leave traces in the form of SiV color centers in the diamond needles.

The normalization of the PL spectra before and after the thermal oxidation using the Raman line (based on the intensity of the diamond peak in our case) allowed for revealing some structural change peculiarities that occurred during the selective oxidation. The luminescent band centered at 650 nm, which was related to the defects at the surface of the diamond, disappeared after the thermal oxidation (see Figure 4 and compare the black and red solid lines). Hence, the thermal oxidation in air was sufficient for removing defective diamond that was located not only in the area between the needles as a whole but also from the surface of the single-crystal diamond needles. Moreover, the intensity of the luminescent band, which was centered at approximately 600 nm and assigned to the NV centers’ phonon-side repetitions, slightly decreased but were still present in the spectra. Hence, this observation indicated the presence of NV centers, both in the nanodiamond matrix and in the diamond needles.

The significant intensity increase of the SiV band (centered at 738 nm) after the thermal oxidation might be related to several complementary processes. One of them was related to the defects associated with silicon that might be additionally activated during thermal oxidation; another process might have been triggered by improvements in the SiV signal collection. It is believed that the PL signal came from the tips of the diamond pyramids [15], which, in the case of the initial sample (before the thermal oxidation), were deep inside of the poorly transmitting film, i.e., under a layer of material that prevented the penetration of both the exciting radiation and the PL signal. This is why it is natural to suppose that removing the blocking layer tremendously improves the SiV signal collection, which was reflected in the PL spectra (see Figure 4 and compare the black line and transparent pink line, whose intense SiV peak made the needles’ Raman signal almost invisible). Moreover, the Raman signal at 1331 cm^−1^ was sensitive to the amount of material and was collected from both a nanodiamond matrix and the diamond needles. Since after oxidation, the nanodiamond matrix was removed, the Raman signal at 1331 cm^−1^ decreased, while the SiV emission was less affected. Thus, normalization relative to the Raman intensity at 1331 cm^−1^ resulted in a higher SiV peak.

The evident similarity of the SEM images obtained for the polycrystalline diamond films manufactured using CVD methods with different types of the gas mixture activation (see, e.g., [32]), including DC-discharge plasma-enhanced CVD [10] and the HF CVD studied in this work, indicates the ability to produce single-crystal needle-like diamonds using different types of CVD.

## 4. Conclusions

Free-standing diamond needles were obtained using a combination of hot-filament CVD and thermal oxidation processes. The obtained diamond crystallites had sharp apexes and smooth basal planes that were terminated by {100} facets. The crystallite length varied from a few hundreds of nanometers to several micrometers, depending on the CVD process duration. A mechanism for the needle-like diamond crystal growth was supposed to be similar to these, which occurred when the polycrystalline diamond films with embedded needles was deposited using the DC-discharge plasma-enhanced CVD method. The proposed approach, which involved hot-filament CVD usage, has the potential for simple and large-scale fabrication of single-crystal diamond needles.

## Data Availability

Data sharing is not applicable to this article.
